# Supplementary material for: Asynchronous magnetic resonance elastography: Shear wave speed reconstruction using noise correlation of incoherent waves
Source: Magn Reson Med. 2022 Oct 27;89(3):990–1001. doi: 10.1002/mrm.29502 (PMC9792433; doi:10.1002/mrm.29502)
Supplement: Supplementary file 1 — DATA S1 MATLAB code used to generate the numerical simulations of Figure 1 [file MRM-89-990-s002.zip › k-Wave/helpfiles/example_pr_2D_tr_line_sensor.html]

2D Time Reversal Reconstruction For A Line Sensor Example (k-Wave)


# 2D Time Reversal Reconstruction For A Line Sensor Example

This example demonstrates the use of k-Wave for the time-reversal reconstruction of a two-dimensional photoacoustic wave-field recorded over a linear array of sensor elements. The sensor data is simulated and then time-reversed using `kspaceFirstOrder2D`. It builds on the 2D FFT Reconstruction For A Line Sensor Example.

- Open the file in the MATLAB Editor
- Run the file in MATLAB

## Contents

- Performing the time-reversal reconstruction
- Comparison with the FFT reconstruction

## Performing the time-reversal reconstruction

The first-order k-Wave functions already described for the simulation of photoacoustic wave propagation can also be used for photoacoustic image reconstruction by assigning the time varying pressure recorded over the detector array to `sensor.time_reversal_boundary_data`. This pressure is then enforced, in time reversed order, as a Dirichlet boundary condition over the given sensor mask. If the sensor mask is given as a set of Cartesian coordinates then the sensor data, indexed as `sensor_data(sensor_position, time)`, must be given in the same order. An equivalent grid-based sensor mask computed using nearest neighbour interpolation is then used to enforce the boundary condition within the computational grid at each time step. If the sensor mask is instead given as a binary grid, the sensor data must be ordered using MATLAB's standard column-wise linear matrix indexing.

By passing the sensor data returned from a k-space forward simulation directly to `sensor.time_reversal_boundary_data` and then calling `kspaceFirstOrder2D`, it is straightforward to simulate the measurement and reconstruction process (note, in this simple example the 'inverse crime' is committed in which the same numerical parameters are used for both simulation and reconstruction). When using the simulation functions in time reversal mode, the array of time points `kgrid.t_array` must be explicitly defined. This array is created here using the `makeTime` method of the `kWaveGrid` class.

```
% run the simulation
sensor_data = kspaceFirstOrder2D(kgrid, medium, source, sensor, input_args{:});

% reset the initial pressure
source.p0 = 0;

% assign the time reversal data
sensor.time_reversal_boundary_data = sensor_data;

% run the time reversal reconstruction
p0_recon = kspaceFirstOrder2D(kgrid, medium, source, sensor, input_args{:});
```

The initial photoacoustic pressure distribution used in the simulation, and the reconstructed initial pressure distribution using time-reversal are shown below.


## Comparison with the FFT reconstruction

It is useful to compare the performance of the time-reversal and FFT reconstruction algorithms. The command line output of the two functions are given below. It is clear that the time-reversal reconstruction takes significantly longer to compute; more than an order of magnitude for this example, even using the slower `'*linear'` interpolation and a non 2^N grid size for the FFT comparison. However, in contrast to the one-step FFT reconstruction, time-reversal can account for a heterogeneous propagation medium, acoustic attenuation, and a sensor mask of arbitrary shape.

```
Running k-Wave simulation...
  start time: 04-Jun-2017 09:00:15
  reference sound speed: 1500m/s
  time reversal mode
  dt: 20ns, t_end: 15.54us, time steps: 778
  input grid size: 88 by 216 grid points (8.8 by 21.6mm)
  maximum supported frequency: 7.5MHz
  expanding computational grid...
  computational grid size: 128 by 256 grid points
  precomputation completed in 0.1263s
  starting time loop...
  estimated simulation time 4.3667s...
  simulation completed in 4.5085s
  total computation time 4.669s
Running k-Wave line reconstruction...
  grid size: 216 by 778 grid points
  interpolation mode: *linear
  applying positivity condition...
  computation completed in 0.062355s
```

A profile through the larger disc, including a comparison with the FFT reconstruction, is also given. The time-reversal reconstruction has a slightly improved signal to noise ratio, although, it is important to note that the inverse crime has been committed.
